# Growth Pattern of Hepatic Metastasis as a Prognostic Index Reflecting Liver Metastasis-Associated Survival in Breast Cancer Liver Metastasis

**DOI:** 10.3390/jcm11102852

**Published:** 2022-05-18

**Authors:** Jieun Lee, Moonhyung Choi, Seungyeon Joe, Kabsoo Shin, Sung-Hak Lee, Ahwon Lee

**Affiliations:** 1 Division of Medical Oncology, Department of Internal Medicine, College of Medicine, Seoul St. Mary’s Hospital, The Catholic University of Korea, Seoul 06591, Korea; befamiliar@catholic.ac.kr (J.L.); kabsoo.shin@catholic.ac.kr (K.S.); 2 Cancer Research Institute, The Catholic University of Korea, Seoul 06591, Korea; 3 Department of Radiology, College of Medicine, Eunpyeong St. Mary’s Hospital, The Catholic University of Korea, Seoul 03312, Korea; choimh1205@gmail.com; 4 Department of Hospital Pathology, College of Medicine, Seoul St. Mary’s Hospital, The Catholic University of Korea, Seoul 06591, Korea; whtmddus0317@naver.com (S.J.); hakjjang@catholic.ac.kr (S.-H.L.)

**Keywords:** breast neoplasm, liver metastasis, hepatic failure, prognosis

## Abstract

Breast cancer with liver metastasis (BCLM) frequently cause hepatic failure owing to extensive liver metastasis compared to other cancers; however, there are no clinicopathologic or radiologic parameters for estimating BCLM prognosis. We analyzed the relationship between radiologic and clinicopathologic characteristics with survival outcomes in BCLM. During 2009–2019, baseline and final abdomen computed tomography or liver magnetic resonance imaging of BCLM patients were reviewed. Liver metastasis patterns were classified as oligometastasis (≤3 metastatic lesions), non-confluent or confluent mass formation, infiltration, and pseudocirrhosis. Thirty-one surgical or biopsy specimens for liver metastasis were immunostained for L1 adhesion molecule (L1CAM), Yes-associated protein 1/Transcriptional co-activator with PDZ-binding motif (YAP/TAZ), and β1-integrin. Out of 156 patients, 77 initially had oligometastasis, 58 had nonconfluent mass formation, 14 had confluent mass formation, and 7 had infiltrative liver metastasis. Confluent or infiltrative liver metastasis showed inferior liver metastasis-associated survival (LMOS) compared to others (*p* = 0.001). Positive staining for L1CAM and YAP/TAZ was associated with inferior survival, and YAP/TAZ was related to final liver metastasis. Initial hepatic metastasis was associated with LMOS, especially confluent mass formation, and infiltrative liver metastasis pattern was associated with poor survival. Positive staining for YAP/TAZ and L1CAM was associated with inferior LMOS, and YAP/TAZ was related to final liver metastasis.

## 1. Introduction

Breast cancer is the most common cancer among women in South Korea and globally [1,2]. Most patients are diagnosed at an early stage based on routine screening by mammography and breast ultrasonography screening; however, metastatic breast cancer (MBC) cases are still reported [3]. The most common metastatic sites of breast cancer are the bones, liver, lungs, and brain [4]. The liver is the third most common metastatic site, with a relatively poorer prognosis than that of other MBCs without liver metastasis [4,5]. Initial breast cancer with liver metastases (BCLM) usually present with oligometastasis with intact liver function but may eventually progress to hepatic failure with extensive infiltrative liver metastasis in end-stage disease [6,7].

The liver is a metastasis-permeable organ that functions through various mechanisms [5,6]. Colorectal cancer (CRC) is the most common solid cancer that metastasizes to the liver. Growth of colorectal cancer with liver metastasis (CRLM) is associated with angiogenesis, which explains, at least partly, why CRLM treatment targets angiogenesis [5,8]. On microscopic examination, CRLM tends to appear as pushing growth patterns with desmoplastic stroma, characterized by newly developed angiogenic microvessels and infiltration of immune cells [9,10,11]. In contrast to CRLM, breast cancer with liver metastasis (BCLM) has a non-angiogenic growth pattern with a preserved hepatic stroma [5,10]. Histopathology of BCLM shows a replacement growth pattern, which indicates the replacement of hepatocytes with breast cancer cells [9]. The mechanism of infiltrative tumor growth of BCLM is based on metastatic tumor cells utilizing pre-existing sinusoidal vessels, defined as vessel co-option [8]. This growth pattern is relatively independent of angiogenesis, with only a few immune cells detected in the infiltrative cancer cells [12].

The etiologies of different histopathologic growth patterns (HGPs) between BCLM and CRLM are poorly understood [9]. During the development of liver metastasis, diverse interactions occur among cancer cells, sinusoidal vessels, hepatocytes, and immune-associated cells, such as Kupffer cells and dendritic cells [5]. There may be differences in the interaction of cancer cells with the liver microenvironment according to the primary origin of cancer cells, but there are limited explanations about the development of distinct HGPs. Previous studies demonstrated that differences in HGPs may be associated with clinical outcomes in liver metastasis. Compared with desmoplastic HGP, replacement HGP shows an inferior response to systemic treatment and is associated with poor outcomes [8,13]. Currently, HGP assessment is solely based on surgical resection and review of the microscopic pathologic features of metastatic lesions. Recently, attempts to identify HGP based on radiologic characteristics have been made in breast cancer and CRC [11,14,15]. Recent studies reported the importance of radiologic images, such as contrast-enhanced computed tomography (CT) and magnetic resonance imaging (MRI), for estimating HGP, mostly in CRLMs. However, only a few studies used radiologic parameters for estimating the clinical course and prognosis of liver metastasis in breast cancer.

Previous studies focused on gene-level analyses that may explain the selective organ-trophism of breast cancer metastasis and prognosis [16,17], but the mechanism underlying the development of replacement HGP in BCLM has not been properly analyzed based on genetic or immunohistochemistry-based studies. BCLM utilizes a non-angiogenic growth mechanism that resembles the growth pattern of brain or lung cancers [18]. The mechanism of infiltrative and vascular co-option tumor growth is reported to be associated with cell adhesion and extravasation [6,8]. In breast cancer, cancer cells may undergo extravasation, adhesion, and vascular co-option during hepatic metastasis. A preclinical model suggested that cancer cells may mimic pericyte behavior during participation in hepatic metastasis, which was previously reported to be associated with the L1 adhesion molecule (L1CAM), Yes-associated protein 1/Transcriptional co-activator with PDZ-binding motif (YAP/TAZ), and the β1-integrin pathway [19].

We aimed to analyze the relationship of radiologic and clinicopathologic characteristics with survival outcomes in BCLM. Particularly, we report about the growth pattern of liver metastasis based on contrast-enhanced CT or MRI findings and its association with BCLM prognosis. We performed a pilot study analyzing immunohistochemical markers (L1CAM, YAP/TAZ, and β1-integrin) possibly associated with the radiologic characteristics and survival of BCLM.

## 2. Materials and Methods

### 2.1. Patients

From January 2009 to May 2019, the medical records of patients with histologically confirmed MBC were retrospectively reviewed in Seoul St. Mary’s Hospital, Catholic University of Korea. Patients diagnosed with liver metastasis based on radiologic studies, such as contrast-enhanced abdominal CT or liver MRI, were enrolled in the study. All patients received systemic treatment for MBC. Other inclusion criteria were as follows: (1) lesions that could be evaluated based on Response Evaluation Criteria in Solid Tumors, version 1.1; (2) Eastern Cooperative Oncology Group performance status of 0–2; and (3) adequate bone marrow and renal function.

This study was approved by the Institutional Review Board (IRB) of Seoul St. Mary’s Hospital, Catholic University of Korea (KC19SESI0001). The information of the medical records of patients were adequately coded and stored as file with security password. The requirement for written informed consent was waived according to the IRB’s decision.

### 2.2. Classification of Liver Metastasis by Radiologic Images

BCLM was diagnosed using iodine contrast-enhanced abdominal CT or gadoxetic acid (Primovist)-enhanced liver MRI. BCLM was classified into five categories based on the number and configuration of the metastatic lesions: oligometastases (≤3 metastatic hepatic lesions with clear margins), nonconfluent metastases (≥4 nonconfluent metastatic hepatic lesions), confluent metastasis (hepatic metastasis with confluent nodules), infiltrative liver metastases (diffuse infiltrative metastases involving bilateral hepatic lobes), and pseudocirrhosis (development of a diffuse nodular contour of the liver with widespread hepatic metastases) [20,21,22,23]. An experienced senior radiologist interpreted and classified patients’ radiologic scans in a blinded manner.

### 2.3. Classification of Molecular Subtype, Immunohistochemical Staining and Specimen Analysis

Hormone receptor-positive breast cancer was defined as an estrogen receptor (ER)-positive or progesterone receptor (PR)-positive cancer according to the American Society of Clinical Oncology-College of American Pathologists guidelines [24]. Molecular subtypes were classified based on 2015 St. Gallen Consensus Conference recommendations [25]: (1) luminal A; (2) luminal B; (3) HER2 positive; and (4) triple-negative breast cancer (TNBC). Luminal A subtype was defined as an ER- and/or PR-positive and HER2-negative result with a Ki-67 index of ≤20%. Luminal B subtype was defined as an ER- and/or PR-positive HER2-negative result with a Ki-67 index of >20%. HER2 positivity was defined as HER2 immunohistochemistry 3 + (circumferential membrane staining: complete, intense, and in >10% of tumor cells) or 2 + (weak-to-moderate complete membrane staining observed in >10% of tumor cells) with HER2 silver in situ hybridization positivity (average *HER2* copy number: ≥6.0 signals/cell) [26], irrespective of the ER or PR status. TNBC was defined as negative ER, PR, and HER2 expression.

Evaluable pathological liver tissue specimens were formalin-fixed and stored in paraffin blocks at Seoul St. Mary’s Hospital, The Catholic University of Korea. Paraffin blocks were serially sectioned into 4 µm thick slices for immunohistochemical staining of L1CAM, YAP/TAZ, and β1-integrin. The primary antibodies used were L1CAM mouse monoclonal antibody (CD171, clone 14.10, 1:100 dilution, SIG-3911, BioLegend, San Diego, CA, USA), YAP/TAZ rabbit monoclonal antibody (D24E4, 1:200 dilution, 8418S; Cell Signaling Technology, Danvers, MA, USA), and β1-integrin rabbit polyclonal antibody (1:200 dilution; GTX 128839; GeneTex). For L1CAM, YAP/TAZ, and β1-integrin staining, tissue sections were deparaffinized with xylene three times for 10 min and rehydrated using 100%, 95%, and 70% ethanol for 5 min each after incubation in an oven at 60 °C for 1 h. Antigen retrieval was performed in a pressure cooker (Electric Pressure Cooker CPC-600; Cuisinart, East Windsor, NJ, USA) for 20 min using 1× citrate buffer (pH 6.0). Endogenous peroxide activity was blocked using methanol-diluted 3% hydrogen peroxide for 15 min. Sections were incubated with the primary antibody β1-integrin for 2 h at 22–25 °C in a humidified chamber. Sections were incubated with the primary antibody, L1CAM, for 2 h at 22–25 °C in a humidified chamber. Sections were incubated with the primary antibody, YAP/TAZ, overnight at 4 °C in a humidified chamber. Sections were subsequently incubated with secondary antibodies (EnVision+System HRP labeled Polymer Anti-rabbit, K4003, DAKO, EnVision+System HRP labeled Polymer Anti-mouse, K4001, DAKO) for 30 min at 22–25 °C. The immunoreaction signal was amplified and measured using a liquid DAB+ Substrate kit (GBI, Bothell, WA, USA). Subsequently, the slides were counterstained with Harris’s hematoxylin (YD Diagnostics, Yongin, Korea).

**Figure 1:**
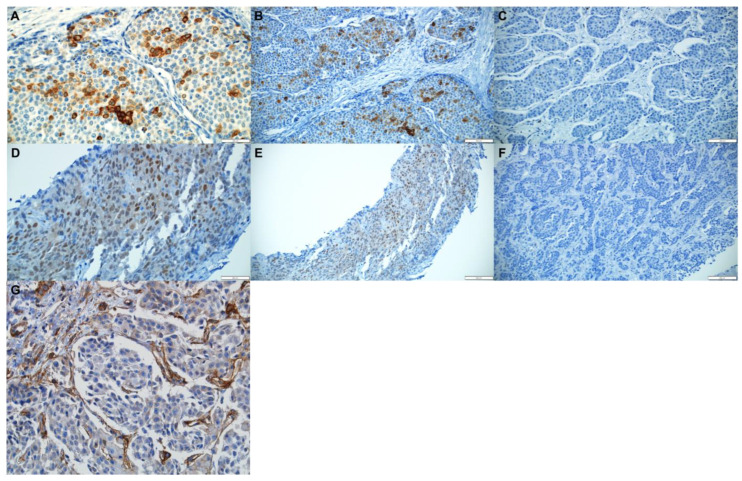
Immunohistochemical staining for L1CAM, YAP/TAZ and β1-integrin in metastatic breast cancer in liver. L1CAM positive (**A**,**B**), L1CAM negative (**C**), YAP/TAZ positive (**D**,**E**), YAP/TAZ negative (**F**), and β1-integrin positive (**G**) ((**A**,**D**,**G**) ×400; (**B**,**C**,**E**,**F**) ×200).

Immunohistochemical evaluation was conducted by a senior pathologist in a blinded manner for patients’ characteristics and outcomes. For L1CAM, the percentage of tumor cells with partial or complete membranous staining was estimated, and cases with more than 5% of membrane-positive tumor cells were considered positive (Figure 1A,B) [27]. For YAP/TAZ, the percentage of tumor cells with nuclear staining was estimated, and cases with more than 5% positive tumor nuclei were considered positive (Figure 1D,E) [28]. For β1-integrin, we estimated the staining intensity of endothelial cells as none, mild, moderate, and severe in both the tumor tissue and normal liver tissue distant from the tumor. If endothelial cells of the tumor tissues were more strongly stained than endothelial cells of normal liver tissues, the case was classified as positive (Figure 1G).

### 2.4. Statistical Analysis

Liver metastasis-associated survival (LMOS) was defined from the initial diagnosis of liver metastasis through radiologic studies conducted on a date near the patient’s death, or during the last follow-up date. Continuous variables are presented as median values, and categorical variables are presented as percentages. Continuous variables were compared using the Mann–Whitney U test, whereas categorical variables were compared using the chi-square test and Fisher’s exact test. Survival analyses were performed using the Kaplan–Meier method and compared using the log-rank test. Hazard ratios (HRs) for LMOS were estimated using a Cox proportional hazards model with a 95% confidence interval (CI). Two-sided *p*-values are presented for all analyses, with *p* < 0.05, which was considered statistically significant. R ver. 3.6.3 (R Foundation for Statistical Computing, Vienna, Austria) was used for all statistical analyses.

## 3. Results

### 3.1. Baseline Patient Characteristics

Between January 2009 and May 2019, 156 patients with breast cancer who were diagnosed with liver metastasis were enrolled. The median follow-up duration in the study was 59.37 months (range: 0.43–161.03 months). Baseline patient characteristics are described in Table 1. Among all patients, 77 (49.4%) initially had oligometastasis, 58 (37.2%) had nonconfluent mass-forming pattern, 14 (8.9%) had confluent mass formation, and 7 (4.4%) had infiltrative liver metastasis. Among all patients, 72 (46.2%) were diagnosed with recurrent or MBC with hepatic metastasis from the initial diagnosis. The initial liver metastasis pattern was significantly associated with the final liver metastasis pattern. When patients were initially diagnosed with liver metastasis, most patients showed normal liver function. Liver function was graded using Child–Pugh score (CPS), which is comprised of five factors: total bilirubin, serum albumin, prothrombin time, and presence of ascites of hepatic encephalopathy. Most of the initially diagnosed BCLM patients were presented with CPS of A. However, liver function deteriorated to CPS B or C as the disease progressed irrespective of systemic treatment. Confluent mass-forming or infiltrative liver metastasis patterns were associated with liver metastasis at initial recurrence or metastasis but with borderline significance. Among patients who initially had confluent mass-forming or infiltrative liver metastasis, more than half of them died from secondary hepatic failure due to extensive liver metastasis. Patients with oligometastasis or nonconfluent mass-forming liver metastasis mostly died of breast cancer progression, aside from hepatic failure.

The percentage of liver metastasis pattern was calculated based on the total number of patients. In each initial liver metastasis column, the percentage of patients was calculated based on the number of patients included in each liver metastasis pattern.

Half of the patients (69 patients, 49.3%) were classified as having luminal B breast cancer. There was no significant association between the molecular subtype and initial or final liver metastasis. Most patients had multiple metastatic sites, including the liver, when diagnosed with liver metastasis irrespective of the molecular subtype (Table 2).

### 3.2. Liver Metastasis-Associated Overall Survival and Associated Factors

**Figure 2:**
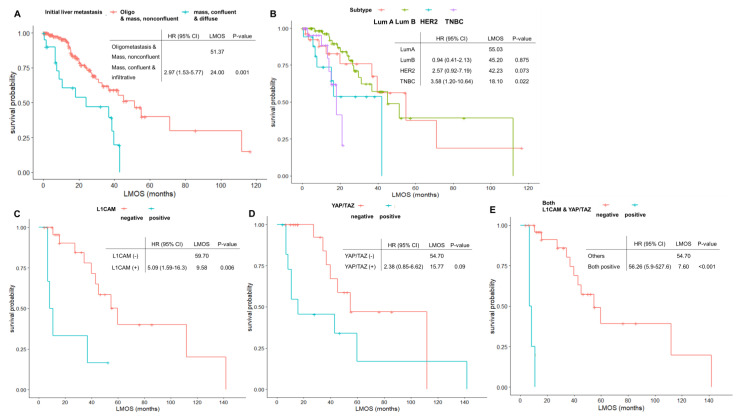
Survival outcome in total patients**.** (**A**) LMOS according to initial radiologic pattern of liver metastasis**;** (**B**) LMOS according to molecular subtypes**;** (**C**) LMOS according to L1CAM expression**;** (**D**) LMOS according to YAP/TAZ expression; (**E**) LMOS according to positive expression for both L1CAM and YAP/TAZ**.** LMOS—liver metastasis-associated overall survival; HR—hazard ratio; CI—confidence interval; L1CAM—L1 adhesion molecule; YAP/TAZ—Yes-associated protein 1/Transcriptional co-activator with PDZ-binding motif**.**

The median LMOS in the total patient population was 42.23 months (range, 0.07–116.67 months). The initial presentation as oligometastasis or nonconfluent mass-forming liver metastasis was associated with superior LMOS compared with confluent mass-forming or infiltrative liver metastasis pattern (median 51.37 months vs. 24.0 months, *p* = 0.001) (Figure 2A). Based on the breast cancer molecular subtype, TNBC was related to inferior LMOS (median 18.10 months, range 0.47–21.63 months) compared with luminal A, B, or HER2-positive subtypes (Figure 2B). A small proportion of patients (14 patients, 9%) underwent metastasectomy or radiofrequency ablation for the local treatment of liver metastasis, which showed no survival benefit in the total patient population (Table 3).

Cox regression analysis was performed for an in-depth analysis of the relationship between clinicopathologic parameters and LMOS. Confluent mass-forming metastasis and infiltrative liver metastasis at initial presentation were associated with inferior LMOS compared with oligometastasis or nonconfluent mass-forming metastasis (HR, 2.97; 95% CI 1.53–5.77; *p* = 0.001). TNBC or HER2-positive breast cancer (BC) also showed poorer LMOS than luminal BC (TNBC, HR 3.58; 95% CI 1.20–10.64, *p* = 0.022; HER2-positive BC, HR 2.57; 95% CI 0.92–7.19; *p* = 0.073). When adjusted for molecular subtypes, the pattern of initial liver metastasis still showed a significant association with LMOS (confluent mass formation or infiltrative liver metastasis, HR 3.38; 95% CI 1.59–7.18; *p* = 0.002) (Table 3).

### 3.3. Expression Pattern of L1CAM, YAP/TAZ, and β1-integrin and Associations with Survival and Liver Metastasis Pattern

In 31 evaluable patients with adequate tissues, the expression patterns of L1CAM, YAP/TAZ, and β1-integrin were analyzed. Although the evaluable sample size was small in number, a positive expression of L1CAM was associated with inferior LMOS when compared with its negative expression (median LMOS 9.58 vs. 59.70 months, *p* = 0.006; Figure 2C). There were trends for shorter LMOS in YAP/TAZ-positive patients, but this was not significant (Figure 2D). Patients who showed positive expression for both L1CAM and YAP/TAZ had inferior survival compared with others (median LMOS 57.70 vs. 7.60 months, *p* < 0.001; Figure 2E). There were no significant differences in LMOS according to β1-integrin expression (data not shown).

Considering that the development and progression of BCLM may be due to adhesion and extravasation of cancer cells to normal sinusoidal vessels, we hypothesized that the expression patterns of L1CAM, YAP/TAZ, and β1-integrin might reflect the gross pattern of BCLM represented with radiologic studies. In fewer cases, positive staining for YAP/TAZ in BCLM cells was associated with the final liver metastasis pattern of evaluable patients (Fisher’s exact test, *p* = 0.02) (Table 4). However, there was no significant association between the hepatic metastasis pattern and staining pattern of L1CAM or β1-integrin.

## 4. Discussion

In the present study, we have shown that the radiologic pattern of liver metastasis may reflect the prognosis of BCLM. We also discovered that positive staining for L1CAM and YAP/TAZ in BCLM cells was related to LMOS, although small in number.

In this study, the baseline metastatic pattern assessed using liver CT or MRI showed a significant association with LMOS. Not all patients with BCLM died from hepatic dysfunction in the study. However, there was a borderline association between baseline liver metastasis pattern and the cause of death. Extensive initial liver metastasis was significantly associated with poor hepatic function, as represented using CPS. There were trends in the relationship between the liver metastasis pattern and the time point of presentation of liver metastasis during the diagnosis of metastatic BC. Based on our analysis, we hypothesized that the initial pattern of liver metastasis may play a role in predicting the clinical course of patients; therefore, clinicians can consider these radiologic findings while selecting treatment options such as chemotherapy regimens.

In addition to the initial liver metastasis pattern, molecular subtype showed a significant association with LMOS in the analysis. Breast cancer molecular subtype is one of the major established prognostic factors in breast cancer. However, the initial liver metastasis pattern still showed a significant association with LMOS when adjusted for molecular subtype in the multivariate analysis. Previous pivotal clinical trials suggest that the presence of liver metastasis is a poor prognostic factor in breast cancer [29]. Not simply applying the presence of liver metastasis as a poor prognostic factor but considering the initial liver metastasis pattern may have a certain role in predicting patient outcomes, which may influence the treatment for BCLM patients.

Previous studies reported that luminal B, HER2-enriched, or ER-expressing BC is associated with liver metastasis [17,30]. However, there are no data reporting the relationship between the radiologic pattern of liver metastasis and BC molecular subtype. Before analyzing the data, we expected that there may be a major association between molecular subtypes. radiologic patterns of BCLM and pathologic features of BCLM. However, we could not analyze the relationship between HGP, molecular subtype, and radiologic findings due to insufficient archival BCLM tissues. Therefore, based on previous report that the radiologic pattern of liver metastasis might reflect the HGP of BCLM [11], we hypothesized that there may be a relationship between BC molecular subtype and radiologic liver metastasis pattern. However, we could not find any significant association between the molecular subtype and pattern of liver metastasis during the analysis. There was an even distribution of initial pattern and final liver metastasis irrespective of the BC molecular subtype. The molecular subtype may not be definitely related to the BCLM pattern, and we can assume that the initiation and progression of BCLM may be associated with other major mechanisms such as tumor cell extravasation, adhesion, proliferation, and angiogenesis related to the peritumoral environment. However, careful interpretation of this result is warranted because classification of the molecular subtype was solely performed based on the Ki-67 index of the primary breast tumor tissue. Further investigation based on primary breast tumor tissues and BCLM tissue is warranted, but there are some limitations based on the rarity of BCLM tissues in routine practice.

Metastasectomy in CRLM is a standard treatment option; however, the results of local treatment such mas metastasectomy or radiofrequency ablation (RFA) for BCLM are controversial [31,32]. Although very few patients were examined, survival of BCLM patients who received local treatment was similar to patients who did not receive local treatment in our analysis. This result may be based on the characteristic HGP of BCLM. As previously mentioned, BCLM tends to grow in a replacement pattern. Breast cancer cells may permeate into the nearby hepatocytes without destruction of the hepatic architecture, and simple resection of BCLM may result in small foci of residual breast cancer cells in the hepatic architecture. Therefore, appropriate systemic treatment needs to be performed in BCLM patients, and local treatment for BCLM should be carefully considered based on a multidisciplinary approach.

As previously mentioned, acquisition of the hepatic tissue from BCLM patients is limited and there are relatively few studies assessing whether the pathologic characteristics of BCLM are reflected in radiologic findings. We hypothesized that the expression pattern of adhesion molecules in hepatic tissues might be associated with the clinical growth pattern of BCLM represented by radiologic findings. We focused on YAP/TAZ as one of the contributing factors for perivascular proliferation, extravasation, and spread of tumor cells in the hepatic environment [33]. A recent study provided evidence that cancer cells may act like pericytes and preform a perivascular niche. YAP/TAZ, L1CAM, and β1-integrin were found to play a certain role during vascular niche co-option, survival, adhesion, and proliferation in multiple organ metastasis including the liver [19,34]. Although a small number of archival tumor tissues were analyzed, we discovered that positive staining for YAP/TAZ in BCLM cells was statistically associated with the radiologic pattern of final liver metastasis. In terms of patients’ survival, positive staining of L1CAM and YAP/TAZ was related to inferior survival outcomes. Considering that YAP/TAZ and L1CAM are related to each other within the common pathway for cell adhesion and proliferation, there might be some relationship among positive staining of YAP/TAZ and L1CAM in BCLM tissues, radiologic pattern of liver metastasis, and survival outcome. For validation, an analysis of the pathologic characteristics, radiologic findings, and immunohistochemical staining pattern of BCLM using a large study cohort is needed.

This study has some limitations. Although the medical records of the enrolled patients were extensively reviewed, there were some missing data in terms of laboratory findings and incomplete assessment of hepatic function as patients showed progression during treatment. Furthermore, there was a limited number of archival BCLM tissues available for analyses, whose results require careful interpretation. However, considering there are relatively few studies focusing on the radiologic and pathologic characteristics of BCLM, our study’s strengths lie in the comprehensive description of the clinical characteristics, survival outcomes, and radiologic as well as pathologic characteristics of BCLM from diverse viewpoints. Recruiting a larger cohort of BCLM patients based on a multicenter study and extending the pathologic analysis at the genetic level may provide more detailed in-depth results in the near future.

## 5. Conclusions

Initial liver metastasis based on radiologic findings may reflect the prognosis of BCLM patients. Infiltrative tumor growth represented in the radiologic study was associated with poor prognosis. In fewer cases, the positive staining of YAP/TAZ in BCLM cells was associated with the final radiologic features of BCLM, and the positive staining of L1CAM was associated with inferior survival. Based on a non-invasive radiologic study, clinicians may predict patient prognosis and therefore select a certain systemic treatment suitable for each patient.

## Figures and Tables

**Table 1:** Baseline characteristics by initial liver metastasis pattern.

	Total	Oligometastasis	Mass, Nonconfluent	Mass, Confluent	Infiltrative	*p* Value
No. of patients	156	77 (49.4%)	58 (37.2%)	14 (8.9%)	7 (4.4%)	
Age (median, interquartile range)	48.0 [42.0;55.0]	51.0 [44.0;58.0]	46.0 [42.0;53.0]	45.5 [39.0;50.0]	47.0 [43.5;51.5]	0.174
Pathology						0.886
Invasive ductal	147 (94.2%)	75 (97.4%)	52 (89.7%)	13 (92.9%)	7 (100.0%)	
Invasive lobular	6 (3.8%)	2 (2.6%)	3 (5.2%)	1 (7.1%)	0 (0.0%)	
Invasive ductal with lobular	1 (0.6%)	0 (0.0%)	1 (1.7%)	0 (0.0%)	0 (0.0%)	
Metaplastic	1 (0.6%)	0 (0.0%)	1 (1.7%)	0 (0.0%)	0 (0.0%)	
Not assessed	0 (0.0%)	0 (0.0%)	1 (1.7%)	0 (0.0%)	0 (0.0%)	
Molecular subtype						0.306
Luminal A	28 (17.9%)	15 (19.5%)	9 (15.5%)	1 (7.1%)	3 (42.9%)	
Luminal B	69 (44.2%)	35 (45.5%)	26 (44.8%)	5 (35.7%)	3 (42.9%)	
HER2 positive	17 (10.9%)	8 (10.4%)	5 (8.6%)	3 (21.4%)	1 (14.3%)	
TNBC	26 (16.7%)	13 (16.9%)	12 (20.7%)	1 (7.1%)	0 (0.0%)	
Not assessed	16 (10.3%)	6 (7.8%)	6 (10.3%)	4 (28.6%)	0 (0.0%)	
Diagnosis of liver metastasis						0.068
initial recurrence or metastasis	72 (46.2%)	32 (41.6%)	25 (43.1%)	9 (64.3%)	6 (85.7%)	
subsequent recurrence or metastasis	84 (53.8%)	45 (58.4%)	33 (56.9%)	5 (35.7%)	1 (14.3%)	
Metastasis site						0.814
liver and others	142 (91.0%)	69 (89.6%)	53 (91.4%)	13 (92.9%)	7 (100.0%)	
liver only	14 (9.0%)	8 (10.4%)	5 (8.6%)	1 (7.1%)	0 (0.0%)	
Final liver metastasis pattern						<0.001
Oligometastasis (≤3)	11 (7.1%)	11 (14.9%)	0 (0.0%)	0 (0.0%)	0 (0.0%)	
mass, nonconfluent	35 (22.4%)	15 (20.3%)	20 (36.4%)	0 (0.0%)	0 (0.0%)	
mass, confluent	48 (30.8%)	28 (37.8%)	16 (29.1%)	4 (33.3%)	0 (0.0%)	
infiltrative	40 (25.6%)	17 (23.0%)	13 (23.6%)	4 (33.3%)	6 (85.7%)	
pseudocirrhosis	14 (8.9%)	3 (4.1%)	6 (10.9%)	4 (33.3%)	1 (14.3%)	
Not assessed	8 (5.1%)					
Initial CPS						<0.001
A	154 (99.4%)	77 (100.0%)	57 (100.0%)	14 (100.0%)	6 (85.7%)	
B	1 (0.6%)	0 (0.0%)	0 (0.0%)	0 (0.0%)	1 (14.3%)	
Last CPS						0.032
A	34 (21.8%)	23 (34.3%)	10 (21.7%)	1 (7.7%)	0 (0.0%)	
B	24 (15.4%)	13 (19.4%)	9 (19.6%)	2 (15.4%)	0 (0.0%)	
C	50 (32.1%)	20 (29.9%)	16 (34.8%)	10 (76.9%)	4 (80.0%)	
Not assessed	23 (14.7%)	11 (16.4%)	11 (23.9%)	0 (0.0%)	1 (20.0%)	
Cause of death						0.08
Cancer	77 (58.8%)	44 (65.7%)	28 (60.9%)	4 (30.8%)	1 (20.0%)	
Hepatic failure due to liver metastasis	46 (35.1%)	18 (26.9%)	15 (32.6%)	9 (69.2%)	4 (80.0%)	
Others	5 (3.8%)	4 (6.0%)	1 (2.2%)	0 (0.0%)	0 (0.0%)	
Not assessed	3 (2.3%)	1 (1.5%)	2 (4.3%)	0 (0.0%)	0 (0.0%)	

HER2—human epidermal growth factor receptor 2; TNBC—triple negative breast cancer; CPS—Child–Pugh score.

**Table 2:** Association between molecular subtype and liver metastasis pattern.

	Luminal A	Luminal B	HER2 Positive	TNBC	*p* Value
No. of patients	28 (20%)	69 (49.3%)	17 (12.1%)	26 (18.6%)	
Initial metastasis					0.505
No	15 (53.6%)	37 (53.6%)	6 (35.3%)	15 (57.7%)	
Yes	13 (46.4%)	32 (46.4%)	11 (64.7%)	11 (42.3%)	
Metastasis site					0.433
liver and others	25 (89.3%)	62 (89.9%)	17 (100.0%)	22 (84.6%)	
liver only	3 (10.7%)	7 (10.1%)	0 (0.0%)	4 (15.4%)	
Initial liver metastasis pattern					0.548
Oligometastasis (≤3)	15 (53.6%)	35 (50.7%)	8 (47.1%)	13 (50.0%)	
mass, nonconfluent	9 (32.1%)	26 (37.7%)	5 (29.4%)	12 (46.2%)	
mass, confluent	1 (3.6%)	5 (7.2%)	3 (17.6%)	1 (3.8%)	
infiltrative	3 (10.7%)	3 (4.3%)	1 (5.9%)	0 (0.0%)	
Final liver metastasis pattern					0.77
Oligometastasis (≤3)	0 (0.0%)	6 (9.0%)	2 (11.8%)	2 (8.7%)	
mass, nonconfluent	7 (26.9%)	16 (23.9%)	5 (29.4%)	5 (21.7%)	
mass, confluent	6 (23.1%)	24 (35.8%)	5 (29.4%)	10 (43.5%)	
infiltrative	9 (34.6%)	17 (25.4%)	4 (23.5%)	4 (17.4%)	
pseudocirrhosis	4 (15.4%)	4 (6.0%)	1 (5.9%)	2 (8.7%)	

HER2—human epidermal growth factor receptor 2; TNBC—triple negative breast cancer.

**Table 3:** Cox-regression analysis of the relationship between clinicopathologic parameters and LMOS.

Characteristics	Liver Metastasis-Associated Overall Survival (LMOS)			
	Univariate Analysis		Multivariate Analysis	
	Hazard Ratio (95% CI)	*p* Value	Hazard Ratio (95% CI)	*p* Value
Molecular subtype				
Luminal B vs. A	0.94 (0.41–2.13)	0.875	0.93 (0.41–2.12)	0.866
HER2 positive vs. A	2.57 (0.92–7.19)	0.073	2.09 (0.73–5.93)	0.165
TNBC vs. A	3.58 (1.20–10.64)	*0.022*	3.47 (1.17–10.3)	*0.025*
Initial liver metastasis				
Yes vs. No	1.24 (0.69–2.25)	0.470		
Metastasis site				
liver only vs. multiple metastasis	0.62 (0.24–1.60)	0.325		
Initial liver metastasis pattern				
mass, confluent or infiltrative vs. oligometastasis or mass, non-confluent	2.97 (1.53–5.77)	*0.001*	3.38 (1.59–7.18)	*0.002*
Local treatment				
Yes vs. No	0.78 (0.36–1.73)	0.545		

HER2—human epidermal growth factor receptor 2; TNBC—triple negative breast cancer.

**Table 4:** Association between immunohistochemical stain of YAP/TAZ and L1CAM with final liver metastasis pattern.

Final Liver Metastasis Pattern	Oligometastasis and Mass, Non-Confluent	Mass, Confluent and Infiltrative and Pseudocirrhosis	*p* Value
YAP/TAZ			*0.020*
negative	10 (32.3%)	9 (29.0%)	
positive	1 (3.2%)	11 (35.5%)	
L1CAM			0.382
negative	10 (32.3%)	15 (48.4%)	
positive	1 (3.2%)	5 (16.1%)	

YAP/TAZ—Yes-associated protein 1/Transcriptional co-activator with PDZ-binding motif L1CAM, L1 adhesion molecule.

## Data Availability

All data are reported in the article.
